# Outcomes and influencing factors of dental implants in fibula, iliac crest, and scapula free flaps: a retrospective case–control study

**DOI:** 10.1186/s40729-024-00522-5

**Published:** 2024-02-09

**Authors:** Marina Kaiser, Simon Burg, Ulrike Speth, Marie-Luise Cotter, Ralf Smeets, Martin Gosau, Daniela König

**Affiliations:** Department of Oral and Maxillofacial Surgery, University Medical Center Hamburg-Eppendorf, Martinistrasse 52, 20246 Hamburg, Germany

**Keywords:** Dental implants, Vascularized free flaps, Oral rehabilitation, Head and neck cancer, Implant survival, Implant success

## Abstract

**Purpose:**

Reconstruction with vascularized bone grafts after ablative surgery and subsequent dental rehabilitation with implants is often challenging; however, it helps improve the patient’s quality of life. This retrospective case–control study aimed to determine the implant survival/success rates in different vascularized bone grafts and potential risk factors.

**Methods:**

Only patients who received implants in free vascularized bone grafts between 2012 and 2020 were included. The free flap donor sites were the fibula, iliac crest, and scapula. The prosthetic restoration had to be completed, and the observation period had to be over one year after implantation. Implant success was defined according to the Health Scale for Dental Implants criteria.

**Results:**

Sixty-two patients with 227 implants were included. The implant survival rate was 86.3% after an average of 48.7 months. The causes of implant loss were peri-implantitis (*n* = 24), insufficient osseointegration (*n* = 1), removal due to tumor recurrence (*n* = 1), and osteoradionecrosis (*n* = 5). Of all implants, 52.4% were classified as successful, 19.8% as compromised, and 27.8% as failed. Removal of osteosynthesis material prior to or concurrent with implant placement resulted in significantly better implant success than material not removed (*p* = 0.035). Localization of the graft in the mandibular region was associated with a significantly better implant survival (*p* = 0.034) and success (*p* = 0.002), also a higher Karnofsky Performance Status Scale score with better implant survival (*p* = 0.014).

**Conclusion:**

Implants placed in vascularized grafts showed acceptable survival rates despite the potential risk factors often present in these patient groups. However, peri-implantitis remains a challenge.

**Graphical abstract:**

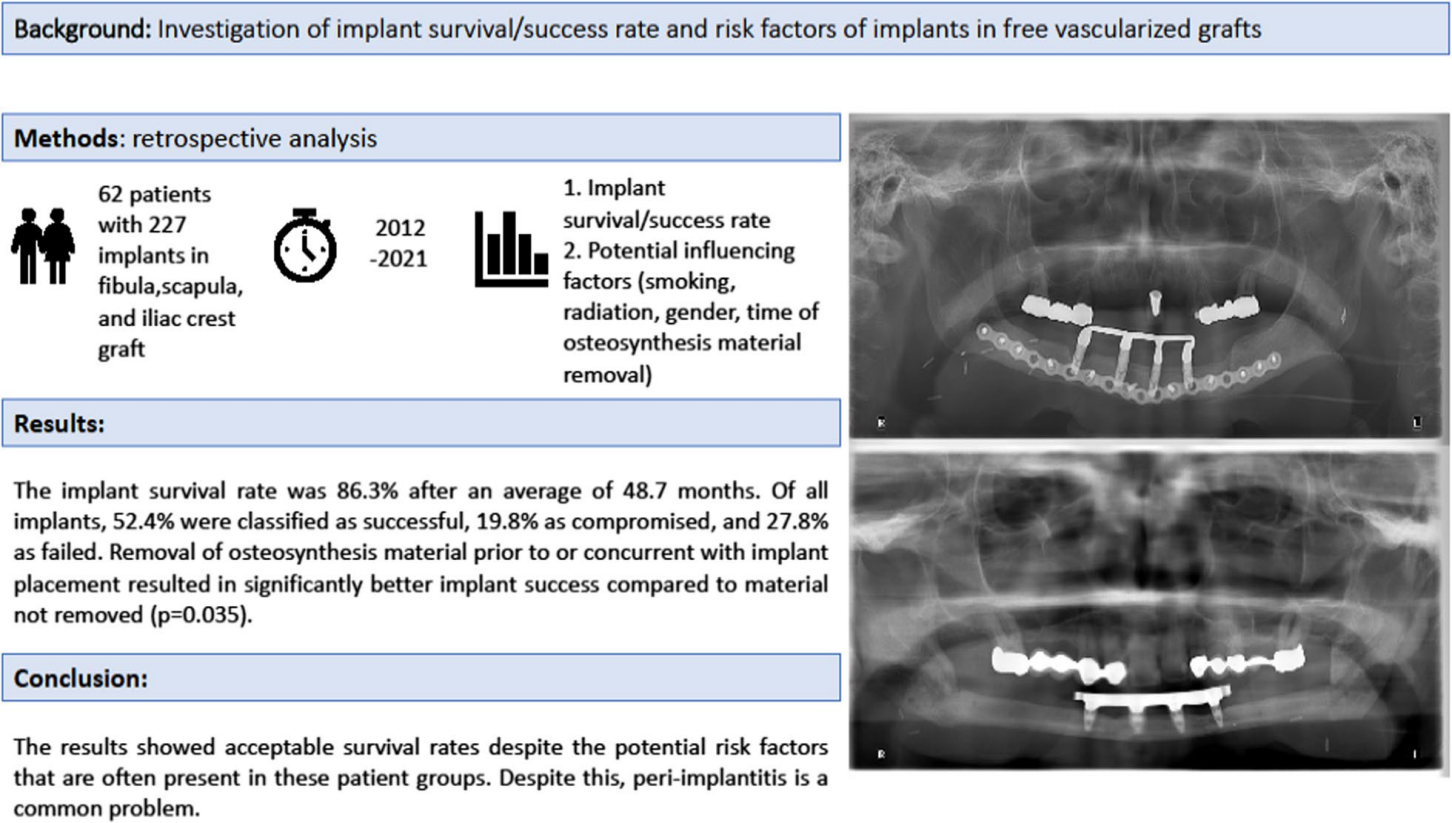

## Background

Resection of tumors, cysts, or osteomyelitis in the head and neck region can result in large compound bone and soft tissue defects and present a major reconstructive challenge. In addition to the development of facial deformities, mastication, articulation, and swallowing are affected [1]. This can result in social and psychological impairments in patients [2]. The emotional resilience of patients in this patient group is also decreased [3].

However, using vascularized free flaps, it is possible to immediately and reliably reconstruct bony defects [2]. Harvesting entities can be the fibula, scapula, iliac crest, rib, or radius [4]. Minor defects can be reconstructed using non-vascularized grafts [5, 6].

Dental implants can be placed into vascularized grafts. This can greatly facilitate prosthetic rehabilitation and significantly improve the quality of life [7, 8]. Conventional, purely mucosa-supported, or tooth-supported prostheses are often unsuccessful in this patient group. This is due to the altered denture foundation with insufficient oral vestibular space and the frequent reduction of potential abutment teeth, resulting in reduced retention [1, 9, 10]. In addition, the loading of sensitive, frequently pre-irradiated tissues with implant-supported prostheses is reduced [3].

Dental implants have been shown to be a safe treatment option for healthy patients, with 10-year survival rates as high as 96.4% [11]. A reduced implant prognosis in free vascularized grafts has been reported in the literature. Ma et al. reported a 5-year cumulative implant survival rate of 81% [10], while other authors described higher 5-year survival rates of 93.6% [12] and 92.2% at three years [8]. Peri-implantitis and mucositis, characterized by swelling, redness, suppuration, and bone loss [13, 14], are commonly reported in implants placed in vascularized bone grafts.

One negative influence may be the reduced soft tissue quality [6, 9, 15]. An adequate zone of keratinized gingiva is essential for healthy peri-implant soft tissues [16]. This has been replaced with resection using osteocutaneous or osteomyocutaneous flaps. Therefore, it is important to perform soft tissue management to restore the appropriate attached areas [6]. These can be vestibuloplasty with either split-thickness skin grafts or free gingival grafts obtained from the palate [6, 15].

Patients with head and neck tumors often smoke and drink alcohol more frequently and have poorer oral hygiene than patients without tumors [17]. Smoking is associated with a higher prevalence of peri-implantitis [18]. Chrcanovic et al. found a 2.23-fold increased risk for implant loss in smokers compared with non-smokers [19]. Some patients also received adjuvant radiotherapy or radiochemotherapy. Radiation impairs bone healing capacity and may cause altered microflora, xerostomia, mucositis and mucosal atrophy [17, 20, 21]. These factors can potentially negatively affect implant outcomes.

The primary aim of this retrospective study is to determine implant survival and success rate in different vascularized grafts. Second, it will be investigated whether different graft types with different bone qualities, localization, patient-specific factors such as age, sex, Karnofsky Performance Status Scale (KPSS), and potential risk factors (smoking, radiotherapy, and chemotherapy) influence implant survival and success. It also investigates whether the timing of osteosynthesis material removal affects implant survival or success. This is based on the assumption that the removal of osteosynthesis material leads to better blood flow and thus improves the implant outcome. To the authors’ knowledge, this has not yet been investigated in comparable studies.

## Materials and methods

All 227 implants investigated in this retrospective case–control study were placed between March 2012 and October 2020 in 62 patients at the Department of Oral and Maxillofacial Surgery of the University Medical Center Hamburg-Eppendorf (UKE). Subsequent prosthetic treatment was performed at the Department of Maxillofacial Surgery, the Department of Prosthodontics of the UKE, or by the patient’s general dentist. Only patients who returned to the Department of Oral and Maxillofacial Surgery for follow-up after prosthetic rehabilitation were included in this study. The following inclusion and exclusion criteria were used:

### Inclusion criteria

- Dental implants of all current manufacturers in free vascularized transplants from the fibula, scapula, or iliac crest.
- Implantation in the UKE.
- Observation time of the patient > 12 months after implantation.

### Exclusion criteria

- No prosthetic treatment.
- Implants outside the free vascularized graft from the fibula, scapula, or iliac crest.

The observation period ended with the last documented examination of implants in the UKE Department of Oral and Maxillofacial Surgery, death of the patient, or implant loss.

### Procedure

After the exclusion of tumor recurrence, well-healed graft, and in irradiated patients, at least six months after the end of the last radiotherapy, prosthetic rehabilitation by implant-supported restorations was planned. The costs were fully reimbursed after prior application to the public health insurance companies. Implant placement was performed under local or intubation anesthesia. The implant system choice depended on the surgeon’s or the prosthodontist’s preference. Implant systems from the following companies were used: Straumann Holding AG (Basel, Switzerland), Camlog Biotechnologies AG (Basel, Switzerland), Bego Implant Systems GmbH and Co. KG (Bremen, Germany), and Dentsply Sirona, Inc. (Charlotte, NC, USA). Postoperatively, depending on the indication, panoramic radiography, digital volume tomography (DVT), or computed tomography (CT) scan was obtained. All implants healed submerged and were bone level implants. After three to four months, implant uncovery followed. Once the soft tissue had healed well after the exposure operation, the impression could be taken, and subsequent implant loading was initiated. The implants were restored using fixed or removable restorations. The following superstructures were used: single crowns, bridges, telescopic crowns, and bar constructions. The osteosynthesis material was partially removed before, simultaneously with, or after implantation. Due to the subjective feeling of better implant survival, the osteosynthesis material was increasingly removed before implantation from 2018 onwards. Soft-tissue management such as vestibulo- and floor-mouth plasty, sometimes in combination with skin or split-thickness skin grafts, was necessary before implant placement, during implant placement, implant uncovery, or after prosthetic restoration to improve soft tissue conditions. Patients were regularly examined for recurrence during tumor follow-up. As part of this, a dental check-up was performed. This occurred four times per year during the first two years. In subsequent years, this occurred twice a year. Radiographs were regularly performed.

### Outcome evaluation

Implant outcomes were measured based on implant survival and success. Implant survival was defined as “the period between implant placement and last examination or implant loss”. Implant success was defined based on the Health Scale for Dental Implants criteria the Pisa Consensus Conference 2007 in Pisa [22]. Misch et al. [22] defined clinical criteria based on which implants are assigned to four groups. These included success (group I), satisfactory survival (group II), compromised survival (group III), and failure (group IV) [22]. Owing to the retrospective design of this study, the criteria of Misch et al. [22] were slightly modified. Groups I and II were merged. Table 1 shows the clinical criteria used in this study and the associated quality levels.

**Table 1:** Classification criteria for implant success/failure oriented to the “Health Scale for Dental Implants” [22]

Implant Quality Scale Group	Clinical conditions
Group I/II: success (optimum health and satisfactory survival)	No pain on function No mobility No exudates history
Group III: compromised survival	May have sensitivity on function Exudates history Peri-implantitis treatment required
Group IV: failure	Pain on function Mobility Radiographic bone loss > ½ length of implant Uncontrolled exudate Unable to be restored Advised explantation No longer in mouth

### Data collection

The medical records of all potential patients were accessed using the patient software Evident (Version 5.73.02.12, Evident GmbH, Bad Kreuznach, Germany) and Soarian (Version 4.5.200, Siemens Healthineers AG, Munich, Germany) and checked for inclusion and exclusion criteria. Patient-specific data were transferred to Microsoft Excel Version 2016 (Microsoft Corporation, Redmond, WA, USA) in anonymized form. Data included age, sex, donor site, localization, reason for graft, radiation, chemotherapy, smoking, KPSS, graft complications, date of implantation, date of osteosynthesis material removal, implant-related criteria, and date of the last examination. Due to the retrospective design of this study, no data on radiation dose/field were available. Furthermore, no statements could be made regarding smoking habits, such as pack years or the number of cigarettes smoked before and after implantation. Previous smokers and those without documentation of smoking status were rated as non-smokers. Available radiographs were used to determine radiographic bone loss using the radiographic software ViewPoint (Version 6.12.2, GE Healthcare, Chicago, IL, USA) and MIM Zero Footprint (Version 6.0, MIM Software Inc., Beachwood, OH, USA). Based on the above criteria, each individual implant was assigned to a grouping, according to Misch et al. [22].

### Statistical analysis

A descriptive analysis of the sample was first performed as part of the statistical analysis. The number and frequency were examined for categorical variables, and for metric variables, the mean values and standard deviations.

In the framework of inferential statistics, the data were exploratively examined for various hypotheses. The type of statistical test was based on the given scale levels of the variables. Contingency tables were calculated using either the Chi-square test or Fisher’s exact test, depending on the underlying distribution. General or generalized linear regressions were used to analyze all other combinations. Due to the exploratory nature of the analysis, no preliminary tests were performed to check the prerequisites of a parametric regression.

*p*-values of *p* < 0.05 are considered to be statistically significant.

The analysis itself was performed in consultation with a biostatistician at the hospital. SAS software version 9.4-M1 was used as the analysis software (SAS Institute, Cary, NC, USA).

## Results

**Fig. 1:**
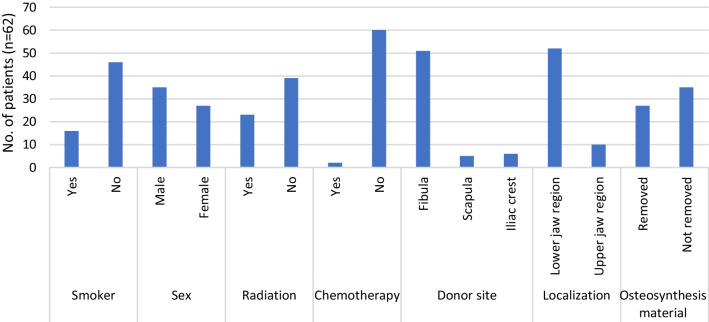
Patient characteristics

In total, data from 328 patients were analyzed. The inclusion and exclusion criteria were met in 62 patients with 227 implants. Implants were placed between March 2012 and October 2020. Twenty-seven patients were women (43.5%), and 35 were men (56.5%). Implantation occurred at an average of 17.9 (range 5–74) months after reconstruction. The mean age at implantation was 58.2 (range 17.0–78.0) years. The mean observation period was 48.7 (range 5–108) months. Additional demographic data and potential risk factors, such as smoking, sex, radio/chemotherapy, donor site, localization, and timing of osteosynthesis material removal, are presented in Fig. 1.

**Fig. 2:**
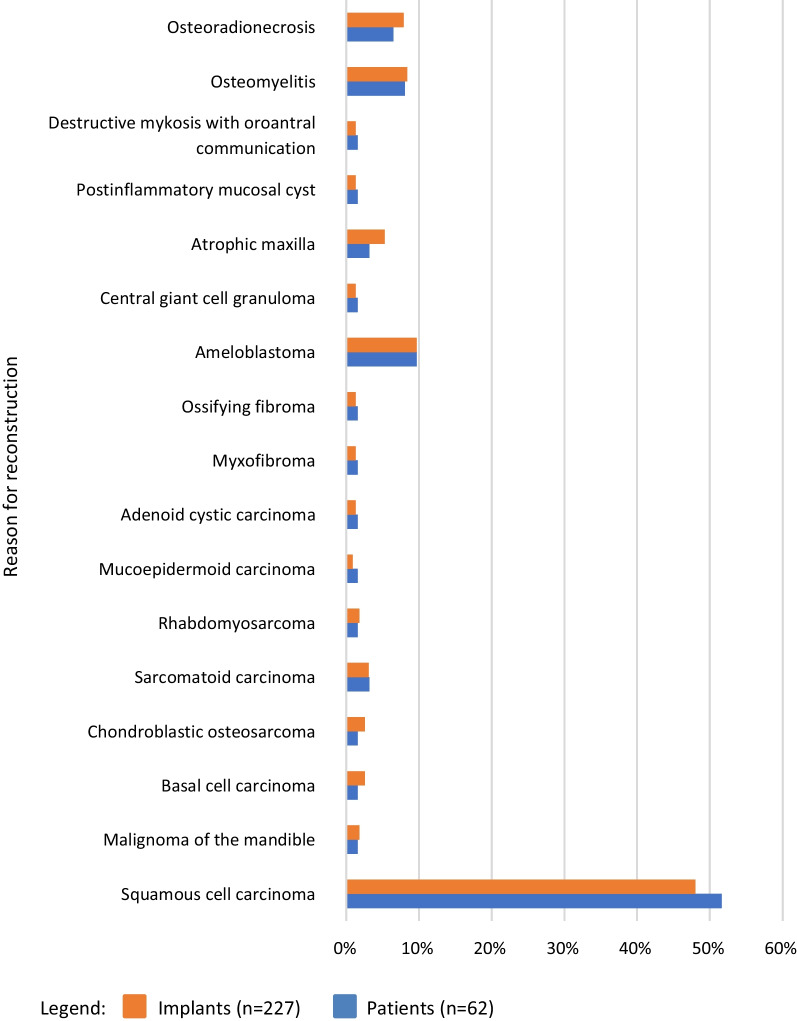
Resection and transplantation reasons

Forty-nine patients (79%) had malignant or benign tumors, resulting in resection and subsequent transplantation. According to the TNM classification of malignant tumors [23], 27 implants were placed in patients with Stage I, 20 with Stage II, 13 with Stage III, and 59 with Stage IVA. The remaining 108 implants were placed in patients without tumors or undocumented tumor stage. The other causes for resection or transplantation are shown in Fig. 2. Four patients had tumor recurrences or lymph node metastasis. This occurred at a mean of 68.79 months after implantation. Secondary carcinomas occurred in five patients (7.9%). These were meningioma (*n* = 1), squamous cell carcinoma (*n* = 2), osteosarcoma (*n* = 1), and esophageal carcinoma (*n* = 1). Complications of the graft included osteoradionecrosis (*n* = 1), pathologic fracture (*n* = 2), and necrosis with pathologic fracture (*n* = 1).

Thirty-one implants (13.7%) were no longer in situ at the end of the observation period in 16 (25.8%) patients. Accordingly, the implant survival rate was 86.3% at 48.7 months. The reasons for implant loss were peri-implantitis (*n* = 24), insufficient osseointegration (*n* = 1), and graft complications such as osteoradionecrosis (*n* = 5) and tumor recurrence (*n* = 1). The average time between implantation and explantation was 35.4 (range 5–82) months.

The implants were classified according to the above-mentioned criteria of the “Health Scale for Dental Implants” of the Pisa Consensus Conference 2007 [22]. The implant success rate was 52.42% after an average of 48.7 months. Of all implants, 52.42% (*n* = 119) were evaluated as successful, 19.82% (*n* = 45) as compromised, and 27.75% (*n* = 63) as failed. The most common reason for classification as compromised or failed was peri-implantitis;34 implants with compromised survival had peri-implantitis. Bone loss was present in 25 implants in over half of the implant body. Twenty-four implants were no longer in situ because of peri-implantitis. Table 2 shows these and other clinical conditions and their frequencies that compromise survival or failure.

**Table 2:** Clinical conditions for compromised survival and failure

Implant Quality Scale Group	Clinical conditions	No. of implants	Percentage (*n* = 277)
Compromised survival	Peri-implantitis treatment required	32	14.1
	Exudates history	2	0.9
	Sensitivity on function	11	4.8
Failure	Radiographic bone loss > ½ length of implant	25	11.0
	Unable to be restored	7	3.1
	No longer in mouth due to peri-implantitis	24	10.6
	Insufficient osseointegration	1	0.4
	Tumor recurrence	1	0.4
	Osteoradionecrosis	5	2.2

Vestibulo- and floor-mouth plasty with split or full-thickness skin was performed for soft-tissue management. Table 3 presents which soft tissue corrections were performed in detail and their frequency. In 98 implants this was done before the prosthetic restoration, in 23 implants after the prosthetic restoration and in 32 implants before and after the prosthetic restoration.

**Table 3:** Frequency of soft tissue corrections

Soft tissue management	No. of implants	Percentage (*n* = 227)
No soft tissue corrections	74	32.6
Closure of oroantral communication	3	1.3
Apically positioned flap	3	1.3
Vestibuloplasty	84	37.0
Vestibuloplasty with full thickness skin	15	6.6
Vestibuloplasty with split thickness skin	22	9.7
Vestibulo- and floor-mouth plasty	22	9.7
Vestibulo- and floor-mouth plasty with full thickness skin	4	1.8

Table 4 shows the number of implant losses and successful, compromised, and failed implants concerning the demographics and risk factors analyzed.

**Table 4:** Patient characteristics and implant outcome

Characteristics	No. of implants (*n* = 227)	Implant loss (*n* = 31) (%)	*p-*value	Success (*n* = 119) (%)	Compromised survival	Failure (*n* = 63) (%)	*p-*value
					(*n* = 45) (%)		
Smoker	60	11 (18.3)	0.219	30 (50.0)	14 (23.3)	16 (26.7)	0.729
Non-smoker	167	20 (12.0)		89 (53.3)	31 (18.6)	47 (28.1)	
Male	129	15 (11.6)	0.307	75 (58.1)	23 (17.8)	31 (24.0)	0.139
Female	98	16 (16.3)		44 (44.9)	22 (22.4)	32 (32.7)	
Radiation	76	10 (13.2)	0.877	48 (63.2)	10 (13.2)	18 (23.7)	0.056
No radiation	151	21 (13.9)		71 (47.0)	35 (23.2)	45 (29.8)	
Fibula	188	26 (13.8)	0.055	104 (55.3)	36 (19.1)	48 (25.5)	0.226
Scapula	20	5 (25.0)		9 (45.0)	5 (25.0)	6 (30.0)	
Iliac crest	19	0 (0.0)		6 (31.6)	4 (21.1)	9 (47.4)	
Lower jaw region	185	21 (11.4)	0.034	105 (56.8)	38 (20.5)	42 (22.7)	0.002
Upper jaw region	42	10 (23.8)		14 (33.3)	7 (16.7)	21 (50.0)	
Osteosynthesis material removed	98	11 (11.2)	0.352	52 (53.1)	25 (25.5)	21 (21.4)	0.035
Not removed	129	20 (15.5)		67 (52.0)	20 (15.5)	42 (32.6)	

Of all implants placed in the maxillary region, 23.8% (10/42) were lost. In the mandibular region, this was 11.4% (21/185). Of all implants placed in the mandibular region, 56.8% were considered successful. In the maxillary region, 33.3% were successful. Implants placed in the mandibular region had significantly better implant survival (*p* = 0.034) and success (*p* = 0.002) than implants placed in the maxillary region (Table 4).

Smoking resulted in worse implant outcomes: 18.3% (11/60) implants were lost in smokers, whereas 12.0% (20/167) were lost in non-smokers. A total of 50.0% (30/60) of the implants in smokers and 53.3% (89/167) of those in non-smokers were considered successful. There was no significant difference in implant survival/success (*p* = 0.22/*p* = 0.73) between smokers and non-smokers. However, smoking resulted in significantly worse implant survival (*p* = 0.0023) and success (*p* = 0.0038) in the maxillary region compared to non-smokers in the maxillary region (Table 5).

**Table 5:** Smoking in the upper jaw region and implant outcome

Implantation in upper jaw region	No. of implants (*n* = 42)	Implant loss (*n* = 10) (%)	*p*-value	Success (*n* = 14) (%)	Compromised survival (*n* = 7) (%)	Failure (*n* = 21) (%)	*p-*value
Smoking	12	7 (58.3)	0.002	0 (0.00)	4 (33.3)	8 (66.7)	0.004
No smoking	30	3 (10.0)		14 (46.7)	3 (10.0)	13 (43.3)	

Radiation (*p* = 0.877/*p* = 0.056) did not significantly affect implant survival/success rates. The mean time to implantation after radiotherapy was 40.7 months (median, 23 months). The minimum was at ten months, and the maximum was at 140 months. The date of irradiation was not documented for seven implants. Implantation performed more than three years after the end of radiotherapy resulted in significantly better implant success (*p* = 0.033) than if the implantation was performed after less than three years (Table 6).

**Table 6:** Time period after radiation and implant outcome

Period after radiation	No. of implants (*n* = 76)	Implant loss (*n* = 10) (%)	*p-*value	Success (*n* = 48) (%)	Compromised survival (*n* = 10) (%)	Failure (*n* = 18) (%)	*p-*value
> 3 years	28	1 (3.6)	0.091	21 (75.0)	5 (17.9)	2 (7.1)	0.033
< 3 years	48	9 (18.8)		27 (56.3)	5 (10.4)	16 (33.3)	

Two patients with a total of ten implants received chemotherapy. Seven implants were classified as successful, three as failure, of which two were no longer in situ at the end of the observation period. Chemotherapy did not significantly affect implant survival (*p* = 0.2) and success (*p* = 0.258).

In patients with previously removed osteosynthesis material, 52 (53.1%) implants were considered successful, 25 (25.5%) as compromised, and 21 (21.4%) as failed. Among the patients in whom the osteosynthesis material was not removed, 67 (51.9%) were rated as implant success, 20 (15.5%) as compromised survival, and 42 (32.6%) as failure. Removing osteosynthesis material before or during implant surgery appears to be associated with better implant success (*p* = 0.035). The effect of osteosynthesis material removal timing on implant survival was insignificant (*p* = 0.352).

Of the implants with KPSS of 80%, 46.2% were no longer in situ, 15.9% with KPSS of 90%, and 10.5% with KPSS of 100% were no longer in situ (Table 7). There was a statistically significant difference in implant survival for implants placed in patients with a higher KPSS score than those with lower scores (*p* = 0.014). There was no significant difference in implant success between the different scores (*p* = 0.88, Table 6).

**Table 7:** KPSS and implant outcome

KPSS (%)	No. of implants (*n* = 95)	Implant loss (%)	*p-*value	Success (%)	Compromised survival (%)	Failure (%)	*p-*value
80	13	46.2	0.014	46.2	0.00	53.8	0.224
90	44	15.9		47.7	25.0	27.2	
100	38	10.5		42.1	26.3	31.6	

Other factors, such as age (*p* = 0.533), sex, and donor site (*p*-values are listed in Table 4), showed no significant influence on implant survival and success.

## Discussion

The aim of this study is to determine the implant survival and success rates of different free vascularized grafts and their limiting factors. The implant survival rate was 86.3%, and the implant success rate was 52.4% after an average of 48.7 months. The main cause of implant loss was peri-implantitis (77.4%). Among all the implants, 52.4% were rated as successful, 19.8% as compromised, and 27.8% as failure. The hypothesis that removing osteosynthesis material would lead to better implant outcomes was confirmed by the implant success rate being significantly better in cases in which osteosynthesis material was previously removed (*p* = 0.035) than in cases in which material was not removed. Implant survival (*p* = 0.034) and implant success (*p* = 0.006) were better at the mandibular site. A higher KPSS (*p* = 0.014) was associated with a significantly higher probability of long-term implant survival. Donor region, smoking, age, radiotherapy and chemotherapy, and sex did not influence implant survival or success.

The implant survival rate is the most frequently used criterion for evaluating implant success [24]. However, it only indicates whether the implant has remained in situ for a specific period. For example, implants that have been advised to be explanted may be incorrectly assessed as still surviving. Therefore, not using them as the sole criteria for evaluating implant outcomes is essential. Implant survival rates in studies that investigated implant survival in various vascularized grafts, as in this study, range from 81 to 93.6% with an observation period of 3–5 years [8, 10, 12, 25], which is consistent with the survival rate determined in this study.

Many studies, even if other criteria were used to evaluate implant success, such as that of Albrektsson et al. [26] reported significantly higher success rates than those in this study [9, 27, 28]. Lodders et al. reported a similar success rate of 58.4% after 5 years [29]. It should be noted that the different studies examined heterogeneous patient groups and used different treatment protocols, which limits the comparability of success rates. It is possible that in other studies, the patients were more strictly selected; for example, Pellegrino et al. [9] did not specify whether any of the selected patients were smokers. Chiapasco et al. [27] applied stricter inclusion criteria. For example, the patients had to have good oral hygiene and no signs of periodontitis.

Peri-implantitis was a frequent problem in this study; it was diagnosed in 17.3% of all surviving implants and caused 77.4% of all implant failures. Kniha et al. reported that free vascularized grafts do not ensure peri-implant bone stability and suffer from significant bone loss within three years [6, 30]. In this study, implant loss after 35.4 months may be because the observation period was at least one year after implant placement, and patients who lost all implants within the first year were excluded. According to the literature, implant loss progresses with time [1, 9, 31]. Stricter follow-up management strategies with annual radiographic examinations and implant cleaning would be helpful in detecting early soft tissue infections in the future. Lodders et al. reported a low rate of implant loss after 5 years of using this approach [29]. Pellegrino et al. showed that the risk of peri-implantitis decreased when pre-prosthetic procedures such as connective tissue grafts or skin grafts were previously performed (18.2% vs. 9.5%) [9]. However, the associated bone loss is problematic in frequent, recurrent procedures involving vestibuloplasty and soft tissue correction [6].

Since most comparable studies used the criteria of Albrektsson et al. [9, 15, 26, 28] to evaluate implant success, this study's implant success rate of 52.4% has limited comparability. The criteria of Albrektsson et al. are the most commonly used and widely accepted criteria for evaluating implant success [22, 26, 32]. In this study, the Health Scale for Dental Implants [22] was used; in contrast to Albrektsson’s criteria, it divides implant success into several quality levels, not only success or failure. Thus, according to Albrektsson et al., implants are already considered a failure in cases of sensitivities and discomfort [26]. However, this is often an accompanying symptom in this patient group after resection. Implants that show increased bone resorption and are stable in the oral cavity are also considered failures, according to Albrektsson et al. [26], but can result in an improved quality of life for the patient. In this study, the original four-level classification, according to Misch et al. [22], was modified because not all criteria proposed by Misch et al. could be identified owing to the retrospective study design. Another limitation is that peri-implant bone loss was not evaluated based on a standardized accurate measurement system because of the lack of radiographic quality and quantity. However, this is not necessary according to Misch et al., but has been done in other comparable studies [22]. Peri-implant bone resorption is best assessed using periapical radiographs [9]. These were only available to a limited extent in this study, as periapical radiographs often cannot be obtained because of the altered anatomy.

This study showed that the KPSS had a significant effect on implant survival. The KPSS is used to grade the limitations of activity, self-determination, and self-care a patient with a malignant tumor experiences [33]. A higher KPSS was associated with a lower probability of implant loss. However, it also showed that implant success was not better with a higher KPSS (*p* = 0.224). The results of this study imply that even patients with a high-stage tumor and in a good general condition can be treated with implants if recurrence can be circumvented.

The choice of donor site often depends on the subjective preferences of the surgeon [34]. The most commonly used free vascularized graft is the vascularized fibula graft [9, 12]. The bicortical bone structure of the fibula provides better primary stability for the implants compared to the iliac crest and scapula [35, 36]. However, after successful osseointegration, no significant differences in the secondary stability of the fibula and iliac crest were found [35]. Our finding that implant outcome is independent of graft type is consistent with other literature [2, 12, 25, 34] and shows that iliac crest and scapular grafts are suitable alternatives. The choice of graft type should always be patient-specific.

Chrcanovic et al. identified a 2.23-fold higher risk of implant loss in smokers than in non-smokers [19]. Studies evaluating implants in vascularized grafts also showed an increased risk of implant loss in smokers [1, 10, 29]. Nicotine affects bone formation and remodeling by affecting osteogenesis and angiogenesis. In addition, nicotine causes vasoconstriction and systemic venoconstruction [19]. Another aspect is that nicotine is anti-inflammatory, and the natural immune response to trauma during implantation is suppressed [20]. In this study, there were significantly lower rates of implant loss and higher rates of implant success in the maxillary region in non-smokers than in smokers. The reason given in the literature is that the effect of local vasoconstriction due to the absorption of nicotine may be higher in the maxilla than in the mandible because the tongue covers the mandibular implants [37, 38]. Based on the results of this study, caution should be exercised, especially with maxillary implants in vascularized grafts in smokers. However, it should be noted that more precise information on smoking habits (pack years and the number of cigarettes smoked before and after implantation) was not provided. Studies have demonstrated that the risk of implant loss increases with the number of cigarettes and pack years [39, 40]

Regarding sex and age, no difference in implant outcome was found in studies involving healthy individuals or patients with vascularized grafts [3, 10, 29, 41]. Thus, the results presented here are consistent with those reported in the literature, although individual authors have reported better implant outcomes in men [17], others in women, and at lower ages [25].

In this study, radiotherapy had no effect on implant survival or success. In contrast, many studies have described a significantly worse implant outcome in individuals subjected to radiation [2, 8, 29]; however, other studies have not shown this [12, 42, 43]. Radiation alters the function of osteoblasts, osteoclasts, and fibrocytes during bone healing and remodeling, adversely affecting bone healing capacity and soft tissue healing [44, 45]. Tissue perfusion decreases, and fibrosis of blood vessels and tissues occurs [44]. This could increase the risks of infection and peri-implantitis. A notable aspect of our study is the long period between the last radiotherapy session and implant placement, averaging 40.72 months. There was a significantly higher implant success rate for implants placed three years after irradiation than for implants placed less than three years after irradiation. This may explain the irradiated implants' better performance compared to the non-irradiated implants. The timing of implant placement with respect to irradiation is the subject of debate; some authors found no significant association between the timing of the last radiotherapy session and implantation [5, 44, 46], whereas others showed a 34% higher implant loss rate for implants placed 6–12 months after radiotherapy and recommended implantation only after one year [47].

Furthermore, a systematic review described higher implant survival after 30 months [5]. This controversy is also shown in physiological studies; the regenerative capacity of the bone is reduced by 70.9% 4 weeks after radiotherapy and increases again by a factor of 2.5 after 1 year [48]. From this, it was deduced that radiotherapy should be started only after 1 year [47]. It is also advantageous to better assess the patients’ anatomical and general conditions to determine likelihood of tumor recurrence. These recurrences most frequently occur after 8–12 months or 2 years [44].

In contrast, Marx and Johnsen showed that tissue fibrosis begins after six months and progresses over time. At the same time, vascularization progressively decreases after six months [49]. However, postponement of prosthetic restoration increases psychological distress in patients [44]. Due to the retrospective study design, no data on the radiation field or dose were available. This represents a limitation of the study, as radiation doses ≥ 50 Gray significantly increased the risk of implant loss [44, 50].

The effect of the timing of osteosynthesis material removal on implant survival and success has not been scientifically studied to the authors’ knowledge. Although some authors have described partial or complete removal by default before or at implant placement [6, 29], others have removed it only when it interferes with implant placement [1, 9]. Other studies excluded this entirely from scientific considerations [51–53]. This study showed a significantly higher rate of implant success for cases in which osteosynthesis material was removed before or at the same time as implant placement. Blood flow may be somewhat permanently decreased when osteosynthesis material is still present. Blood flow plays an important role in bone remodeling processes: oxygen, nutrients, and mediators are transported to bone tissue. These mediators regulate the interaction of cells during bone remodeling processes [19]. The drilled holes and the compression pressure of the osteosynthesis plates initially reduce the periosteal blood supply. However, this does not extend beyond a critical level, and thus allows the graft to integrate [54]. The cortical bone of the diaphysis of the fibula is supplied by periosteal and medullary vessels [55]. Periosteal blood supply is strongly expressed in the fibula, whereas endosteal blood supply is not essential for graft survival [56]. Porcine studies of osteotomized and plated fibulas showed that blood flow decreased significantly postoperatively when compared with the control group [55]. Microangiographic studies by Rhinelander demonstrated that cortical blood flow in the canine tibia was decreased in the area of the osteosynthesis plates even six weeks after insertion. In areas where the plates fell out randomly, the blood supply in the cortex increased again [57]. However, further animal studies showed that after immediate decreases in blood flow after osteosynthesis, blood flow increased to previous levels 2.5 months post-op [58, 59]. Since these are in vitro studies, it is questionable whether this mechanism also applies in vivo or whether cofactors, such as smoking and radiotherapy, permanently reduce blood flow. It is also questionable whether the osteosynthesis material also absorbs and transmits acting forces after osseointegration of the implants and thus reduces functional remodeling: Uhthoff and Finnegan showed through animal experiments on the femur of dogs that the permanent presence of osteosynthesis plates leads to a reduction in bone mass and delayed remodeling. If plates were removed early, structured remodeling and the return to average bone mass could be realized [60]. With the osteosynthesis material still present, there is also a risk of spontaneous exposure to the material. This can create a portal of entry for bacteria and increase the risk of osteomyelitis. This risk is minimized by removing the osteosynthesis material.

Therefore, although we do not know the exact pathogenesis of the various possible explanations, the data of this study advocate the removal of the osteosynthesis material. Further prospective randomized studies are required in this regard.

## Conclusion

Implantation in free vascularized grafts is a safe procedure, as noted by the low graft complication rate and the acceptable survival rate despite the potential risk factors for this group of patients. However, peri-implantitis is a severe complication. Closer clinical and radiological monitoring is required to detect and treat it at an early stage. The possible positive influence of osteosynthesis material removal should be investigated in future randomized prospective studies. Removing osteosynthesis material prior to or at the time of implant placement is a simple and practical procedure to improve implant success.

## Data Availability

The datasets used and/or analyzed during the current study are available from the corresponding author on reasonable request.

## Abbreviations

CT scan: Computed tomography scan
DVT: Digital volume tomography
ICOI: International Congress of Oral Implantologists
KPSS: Karnofsky Performance Status Scale
UKE: University Medical Center Hamburg-Eppendorf
